# Nmnat2 attenuates amyloidogenesis and up-regulates ADAM10 in AMPK activity-dependent manner

**DOI:** 10.18632/aging.203634

**Published:** 2021-10-13

**Authors:** Xiang-Shu Cheng, Fang-Xiao Shi, Kun-Peng Zhao, Wang Lin, Xiao-Ying Li, Jun Zhang, Yao-Yao Bu, Rui Zhu, Xiao-Hong Li, Dong-Xiao Duan, Xin-Ying Ji, Jian-She Wei, Jian-Zhi Wang, Jin Du, Xin-Wen Zhou

**Affiliations:** 1 Department of Neurology, Translational Medicine Center, Huaihe Hospital Affiliated to Henan University, Kaifeng 475000, Henan, China; 2 Department of Pathophysiology, Key Laboratory of Neurological Disease of National Education Ministry and Hubei Province, Institute for Brain Research, School of Basic Medicine, Tongji Medical College, Huazhong University of Science and Technology, Wuhan 430030, Hubei, China; 3 Department of Respiratory, Huaihe Hospital Affiliated to Henan University, Kaifeng 475000, Henan, China; 4 Brain Research Laboratory, Henan University, Kaifeng 475004, Henan, China; 5 Department of Physiology, Basic Medical College, Zhengzhou University, Zhengzhou 450001, Henan, China; 6 Department of Microbiology, Henan International Joint Laboratory for Nuclear Protein Regulation, School of Basic Medical Sciences, Henan University, Kaifeng 475004, Henan, China; 7 Department of Psychiatry, Henan Key Lab of Biological Psychiatry, Henan Mental Hospital, The Second Affiliated Hospital of Xinxiang Medical University, Xinxiang 453002, Henan, China

**Keywords:** Alzheimer disease, nicotinamide mononucleotide adenylyltransferase 2, AMPK, ADAM10, amyloid-β

## Abstract

Amyloid-β (Aβ) accumulating is considered as a causative factor for formation of senile plaque in Alzheimer’s disease (AD), but its mechanism is still elusive. The Nicotinamide mononucleotide adenylyltransferase 2 (Nmnat2), a key redox cofactor for energy metabolism, is reduced in AD. Accumulative evidence has shown that the decrease of α-secretase activity, a disintegrin and metalloprotease domain 10 (ADAM10), is responsible for the increase of Aβ productions in AD patient’s brain. Here, we observe that the activity of α-secretase ADAM10 and levels of Nmnat2 are significantly decreased, meanwhile there is a simultaneous elevation of Aβ in Tg2576 mice. Over-expression of Nmnat2 increases the mRNA expression of α-secretase ADAM10 and its activity and inhibits Aβ production in N2a/APPswe cells, which can be abolished by Compound C, an AMPK antagonist, suggesting that AMPK is involved in over-expression of Nmnat2 against Aβ production. The further assays demonstrate that Nmnat2 activates AMPK by up-regulating the ratio of NAD^+^/NADH, moreover AMPK agonist AICAR can also increase ADAM10 activity and reduces Aβ1-40/1-42. Taken together, Nmnat2 suppresses Aβ production and up-regulates ADAM10 in AMPK activity-dependent manner, suggesting that Nmnat2 may serve as a new potential target in arresting AD.

## INTRODUCTION

Alzheimer’s disease (AD) is the most prevalent neurodegenerative disorder affecting more than 44 million patients worldwide [1]. It is characterized as a slow loss of cognitive function, leading to dementia and death [2]. The neuropathologic hallmarks of AD include extracellular protein deposits of amyloid plaques and intracellular phosphorylation and tangles of neurofibrillar tau protein [3, 4]. Amyloid-beta (Aβ), a 38-42 amino acid long peptide, is prone to aggregation and forms the major constituent of plaques [5, 6]. Aβ peptides and its pathogenic aggregations initiate a cascade of widespread protein depositions and induce neurodegeneration diseases, such as AD [7, 8]. Aβ is generated from the sequential proteolytic processing of amyloid precursor protein (APP) by the enzymes β- and γ-secretase [9, 10]. Indeed, Aβ production can be avoided by an alternate APP cleavage pathway mediated by the α-secretases in the normal brain [11]. Interestingly, α-secretase cleavage of APP generates a secreted form of APP (sAPP), which has been shown to play a role of neurotrophy and neuroprotection in AD models [12, 13].

A disintegrin and metalloprotease (ADAM) is a multi-functional gene family including ADAM9, ADAM10 and ADAM17, which can act as α-secretase in various cell lines [14, 15]. ADAM10 is particularly expressed in mouse and human brain, and is the physiologically essential constitutive α-secretase cleavage of APP, but not ADAM9 or 17 [16–18]. The up-regulation of ADAM10 in AD model mice prevents formation of Aβ plaques and cognitive deficits [19, 20]. It is reported that ADAM10 is reduced in AD brain or in cerebrospinal fluid (CSF) and platelets from AD patients [21–23], but the upstream effectors of regulating α-secretase ADAM10 are still not fully ascertained. Therefore, to explore upstream targets of regulating α-secretase ADAM10 are valuable for developing drugs to reduce Aβ and cure AD.

Nicotinamide mononucleotide adenylyltransferase (Nmnat) is a vital enzyme in catalyzing the synthesis of Nicotinamide adenine dinucleotide (NAD^+^) from NMN [24, 25]. There are three Nmnat isoforms including Nmnat1, Nmnat2 and Nmnat3, which have been identified in human and mouse brains [26–28]. Compared with Nmnat1 and Nmnat3, Nmnat2 is specifically and highly expressed in brain [29, 30], and over-expression of Nmnat2 shows a neuroprotective effect in AD models of *Drosophila* and rodents [31–34]. Indeed, accumulative evidence has shown that mRNA of Nmnat2 is reduced in the brains of AD patients and mouse models of AD [35–37]. All of previous research suggest that Nmnat2 is involved in AD development, but its underlying mechanism is not fully elucidated.

Here, we find that the accumulation of Aβ increases in parallel with reduction of ADAM10 in Tg2576 mice. The over-expression of Nmnat2 suppresses amyloidogenesis and activates ADAM10 in AMPK activity-dependent manner in N2a/APPswe cells, and Nmnat2 activates AMPK through increasing the ratio of NAD^+^/NADH.

## MATERIALS AND METHODS

### Reagents and antibodies

NAD/NADH assay kit (Colorimetric) was purchased from Abcam. Bicinchoninic acid (BCA) protein detection kit, chemiluminescent substrate kit, and phosphocellulose units were from Pierce. Diaminobenzidine (DAB), and Hoechst 332621 were from Sigma-Aldrich. Lipofectamine 2000 was from Invitrogen. OPTIMUM and other reagents for cell culture were from Gibco. AICAR (5-aminoimidazole-4-carboxamide-1-β-riboside) was from Cell Signaling, and Compound C was from Millipore. Plasmid Flag-Nmnat2 was kindly gifted by Dr. Michael P. Coleman (The Babraham Institute, Babraham Research Campus, Cambridge, United Kingdom). The target sequences (GeneBanK NM_175460) for mouse Nmnat2 messenger RNA (mRNA) is 5′-GCACAAGACTGGAAGATTT-3′. The scrambled siRNA sequence is 5′-TTCTCCGAACGTGTCACGT-3′. The Nmnat2 siRNA and scrambled siRNA are inserted into pMagic4.1 vector to generate Nmnat2-siRNA-EGFP (siNmnat2) and scramble-siRNA-EGFP plasmids. Some antibodies are as follows: Nmnat2 (Abcam), 22C11 (Millipore), p-APP (Thr-668) (Biosource), ADAM10 (Abcam), G2-10 (Millipore), G2-11 (Millipore), 6E10 (Millipore), 4G8 (Millipore), Y188 (Abcam), AMPK (Sigma), p-AMPK (Thr172) (Abcam), DM1A (Abcam), β-actin (Abcam). See Table 1 for more details.

**Table 1:** Antibodies used in the study.

**Antibody**	**Upload (μg)**	**Specificity**	**Source (Type)**	**Dilution**			**Company**
				**WB**	**IHC(IF)**	**ELISA**	
**Nmnat2**	30	Total Nmnat2	Mouse	1:500	1:200		Abcam
**22C11**	20	Total APP	Mouse	1:500			Millipore
**p-APP**	20	p-APP at Thr668	Rabbit	1:1000			Biosource
**ADAM10**	30	ADAM10	Rabbit	1:500			Abcam
**G2-10**	-	Anti-Aβ40	Mouse			1:1000	Millipore
**G2-11**	-	Anti-Aβ42	Mouse			1:1000	Millipore
**6E10**	40	Aβ1–16	Mouse	1:500	1:200		Millipore
**4G8**	40	Aβ17–24	Mouse	1:500			Millipore
**Y188**	30	Anti-α/β-CTF	Mouse	1:1000			Abcam
**AMPK**	20	Total AMPK	Rabbit	1:1000	1:200		Sigma
**p-AMPK**	25	p-AMPK at THR172	Rabbit	1:500			Abcam
**DM1A**	10	α-Tubulin	Mouse	1:1000			Abcam
**β-actin**	10	β-actin	Mouse	1:1000			Abcam

Key: IHC, immunohistochemisty; IF, immunofluorescence; WB, western blot.

### Cell culture, transfection and drug treatment

N2a cells stably transfected with human APP_695_ harboring the Swedish double mutation (N2a/APPswe) were provided by Dr. Jin-Jing Pei (Karolinska Institutet, Department of Neurobiology, KI-Alzheimer Disease Research Center, Novum, Huddinge Sweden). N2a/APPswe were grown in 1:1 DMEM (Dulbecco’s modified eagle’s medium) / Opti-MEM supplemented with 10% fetal bovine serum (FBS), penicillin, streptomycin, and 200 μg/ml G418, and then maintained in a humid atmosphere containing 5% CO_2_. Cells were tested negative for mycoplasma contaminants. For plasmid transfection, the N2a/APPswe cells were grown in 6-well plates at confluence 70-80%, and then cells were transiently transfected with Nmnat2-Flag plasmid (Nmnat2) or Nmnat2-siRNA-EGFP plasmid cDNAs (siNmnat2) using Lipofectamine 2000 reagent according to the manufacturer’s instructions. For 2mM AICAR or 20 μm Compound C drug treatments, N2a/APPswe cells transfected Nmnat2 cDNAs were treated at confluence for the indicated concentrations and incubation times. Medium was then changed, and treatments were continued for another twenty four hour, and then harvested cells.

### Animal

Tg2576 mice were purchased from Jackson Laboratory (Bar Harbor, ME, USA). These mice over-express human APP695 with a double mutation KM670/671NL. All mice were produced by the Experimental Animal Center of Tongji Medical College, Huazhong University of Science and Technology. The genotype was confirmed by PCR analysis of tail biopsies. The mice were housed with free access to water and food under a 12:12 hr reversed light-dark cycle with light on at 8:00 pm after weaning. All animal (male) experiments were performed according to the “Policies on the Use of Animals and Humans in Neuroscience Research” revised and approved by the Society for Neuroscience in 1995, and the animal study was approved by the Academic Review Board of Tongji Medical College.

### Immunofluorescence and ELISA

Immunofluorescence and ELISA were carried out according to the procedure described previously [38–40]. For cell studies, N2a/APPswe cells were cultured on coverslips and fixed with 4% paraformaldehyde. Rhodamine red-X- or Oregon Green 488-conjugated secondary antibodies (Invitrogen, Carlsbad, CA, USA) were used for immunofluorescence. The images were visualized using a laser two-photon confocal microscope (LSM510, Zeiss, Oberkochen, Germany).

The levels of Aβ in the medium of N2a/APPswe cells transfected with Nmnat2 or CC treatment were measured by a sandwich ELISA kit using an anti-Aβ N-terminal antibody and an anti-Aβ1-40 or Aβ1-42 C-terminal antibody, according to the manufacturer’s instructions (Biosource International, Camarillo, CA, USA).

### Western blot

Western blot was performed according to the established methods in our laboratory [41, 42]. In brief, the cortex was rapidly removed from brain and homogenized on ice using a Teflon glass homogenizer in the buffer containing 50 mM Tris-HCl, pH 7.4, 1% NP-40, 0.25% Na-deoxycholate, 150 mM NaCl, 1 mM Na_3_VO_4_, 1 mM EDTA, 50 mM NaF, 1 mM PMSF and protease inhibitors mixture (2 mg/l each of aprotinin, leupeptin and pepstain A). The homogenates were mixed with loading buffer (3:1, v/v) containing Tris-HCl (pH 7.6) 200 mM, 8% sodium dodecyl sulfate (SDS), 40% glycerol, 40 mM dithiothreitol (DTT) and boiled for ten minutes. For cell experiment, the cells were rinsed twice with ice-cold phosphate-buffered saline (PBS, pH 7.4), then lysed in a cooled buffer containing 50 mM Tris-Cl, pH 8.0, 150 mM NaCl, 1% NP-40, 0.5% Na-deoxycholate, 0.1% SDS, 0.02% NaN3, 100 μg/ml PMSF, and 10 μg/ml protease inhibitors (leupeptin, aprotinin, and pepstatin) followed by sonication for five seconds on ice. The cell lysates were added to one-third volume of sample buffer containing Tris-HCl (pH 7.6) 200 mM, 8% sodium dodecyl sulfate (SDS), 40% glycerol, 40 mM dithiothreitol (DTT) and boiled in a water bath for ten minutes. The protein concentrations of all samples were measured by BCA kit (Pierce, Rockford, IL, USA) according to the manufacturer’s instruction. For sAPPα detection, the medium of N2a/APPswe cells was collected, cleared by centrifugation, lyophilized, and finally resuspended in the SDS-sample buffer. The equal amounts of proteins were separated by 10% SDS-polyacrylamide gel electrophoresis and transferred to nitrocellulose (NC) membranes. The membranes were blocked with 5% non-fat milk dissolved in TBSTween-20 (50 mM Tris HCl, pH 7.6, 150 mM NaCl, 0.2% Tween-20) for one hour and probed with primary antibody Nmnat2 (1:500), 22C11 (1:500), p-APP (1:1000), ADAM10 (1:1000), 6E10 (1:500), 4G8 (1:500), Y188 (1:1000), AMPK (1:1000), p-AMPK (Thr172) (1:500), DM1A (1:1000), and β-actin (1:1000) at 4° C overnight. The blots were detected using anti-rabbit or anti-mouse IgG secondary antibody conjugated to IRDyeTM (800CW, Licor Biosciences, Lincoln, NE, USA) at room temperature for one hour and visualized by infrared fluorescence imaging. The optical density of bands was quantified by Odyssey system (Li-Cor Bioscience, Lincoln, NE, USA). Proteins were normalized against DM1A, see Table 1 for more details.

### Real-time quantitative PCR

Total cortex RNA was extracted using TRIzol reagents according to the instructions (Invitrogen, Carlsbad, CA, USA) and reverse-transcribed to cDNA using reverse transcription reagents kit (Takara, Dalian, China). Fifty nanograms of cDNA were used for real-time PCR. Primers for ADAM10 were 5’-CTGGCCAACCTATTTGTGGAA-3’ and 5’-GACCTTGACTTGGACTGCACTG-3’, and primers for GAPDH were 5’-AACGACCCCTTCATTGAC-3’ and 5’- TCCACGACATACTCAGCAC -3’, respectively. The parameters of PCR cycle were 95° C/10 min, and 40 cycles of 95° C/10 s, 60° C/30 s, and 72° C/30 s. The amplification and analysis were performed using a StepOne Plus Real-Time PCR Detection System (Life Technologies, Grand Island, NY, USA). Samples were compared using the relative CT method.

### NAD^+^/NADH quantification assay

NAD^+^/NADH ratio was calculated from the cortex and N2a/APPswe cells using NAD^+^/NADH assay kit provided by the Abcam. In brief, the cortex from 10 month old Tg2576 mice and N2a/APPswe cells transiently transfected with Nmnat2 plasmid cDNAs were homogenized in ice cold NAD^+^/NADH extraction buffer (freeze/thaw two cycles for cells) after the samples washed with cold PBS, and then centrifuged at 14000 rpm for 5 min. The supernatants were filtered through 10 kD molecular weight cutoff filters at 4° C to remove the enzymes that consume NADH rapidly, separately. 50 μl of ultra filtrates were heated for 30 min at 60° C in a water bath to decompose NAD^+^ for NADH measurement because NADH will still be intact under these conditions. The heated and unheated samples were mixed separately with NAD^+^/NADH cycling mix assay for 5 min in a labeled 96-well plate in duplicates. The color was developing with NADH developer solution, and the absorbance was measured at 450 nm after 2 hour. We measured the protein content of an aliquot of homogenized samples before ultrafiltration by using standard Biorad procedure. The concentrations of NAD^+^ and NADH were expressed in pmol/10^6^ cells or ng/mg protein based on standard NADH readings.

### Statistical analysis

All data were presented with the mean *±* S.D. and analyzed using SPSS 12.0 statistical software (SPSS Inc., Chicago, IL, USA). The one-way analysis of variance (ANOVA) procedure followed by LSD’s post hoc tests and student *t* test were used to determine the differences among groups. *P <* 0.05 was accepted as statistically significant.

## RESULTS

### Decrease of Nmnat2 and ADAM10 is associated with amyloidogenesis in Tg2576 AD mice model

**Figure 1:**
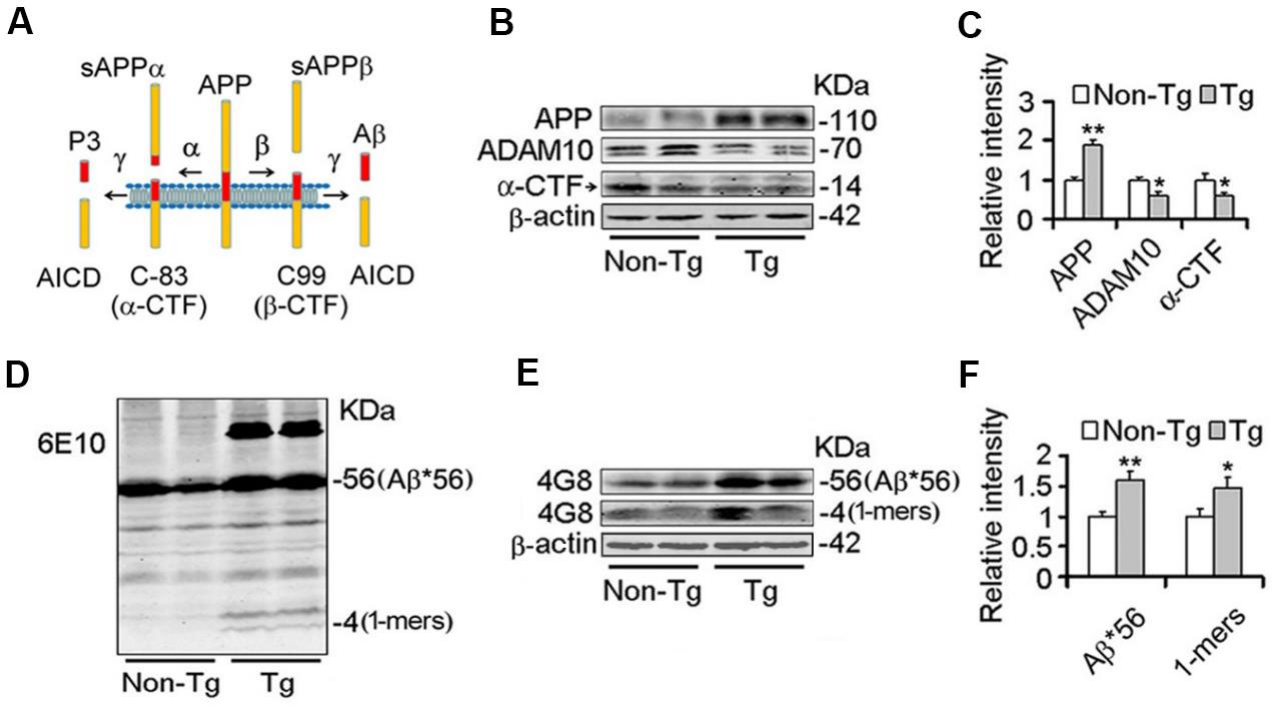
**Decrease of ADAM10 is simultaneous with the increase of Aβ productions in the cortex of 10-month old Tg2576 mice.** (**A**) The scheme of the proteolytic processing of APP. Aβ, amyloid-β peptide with 40 (Aβ1-40), or 42 amino acid residues (Aβ1-42); APP, amyloid precursor protein; AICD, APP intracellular domain; APPα, soluble APP after α-secretase cleavage (α-fragment); APPβ, soluble APP after β-secretase cleavage; C99, C-terminal fragment of APP of 99 amino acids after β-secretase cleavage (β-CTF); C83, C-terminal fragment of APP of 83 amino acids after α-secretase cleavage (α-CTF); P3, N-terminal fragment of C83 after α-secretase cleavage. (**B**–**F**) The cortex extracts were prepared from 10 months old Tg2576 mice (Tg) and the age-matched littermates (Non-Tg), then the levels of ADAM10, α-CTF, and Aβ productions were measured by western blot (**B**, **D**, **E**) and quantitative analysis (**C**, **F**) (n=5 for each group). The data were expressed as means ± S.D. *P < 0.05, **P < 0.01 vs control.

Studies have demonstrated that Nmnats play an important role in maintaining axonal, dendritic and neuronal integrity [43–45], and over-expression of Nmnats offer several protective effects against neurodegeneration and axonal degeneration [31–33]. Our previous results demonstrated that Nmnat2 attenuates tau phosphorylation through activation of PP2A [35]. ADAM10 is the valid active component of α-secretase [15, 16]. Up-regulation of ADAM10 in AD mouse models prevents the formation of senile plaques and cognitive deficits [11, 46, 47]. The levels of ADAM10 and Aβ were detected by western blot and immunohistochemistry in 10-month old Tg2576 mice and the age-matched wild type littermates. We observed that the ADAM10 levels (Figure 1B, 1C) and cleavage of APP generating α-CTF levels (Figure 1A–1C) were decreased significantly, suggesting that activity of α-secretase reduced in Tg2576 mice. Meanwhile the Aβ productions were increased obviously in particular soluble Aβ and its oligomers (Figure 1D–1F). Importantly, we found that a 56-kDa soluble dodecameric Aβ (Aβ^*^56-kDa) is significantly enhanced, which is supposed to disrupt memory in middle-aged Tg2576 mice [48], (Figure 1D–1F). The soluble Aβ oligomers, containing Aβ^*^56-kDa, impair synapse structure and function and cause memory deficits in Tg2576 mice and AD brains [7, 8, 49, 50]. Our previous study found that Nmnat2 levels are obviously decreased in the cortex and hippocampus in 10-month old Tg2576 mice compared with the age-matched wild type littermates [35]. All data indicated that the decrease of Nmnat2 and ADAM10 is associated with amyloidogenesis in Tg2576 mice.

### Over-expression of Nmnat2 suppresses Aβ production and up-regulates ADAM10

**Figure 2:**
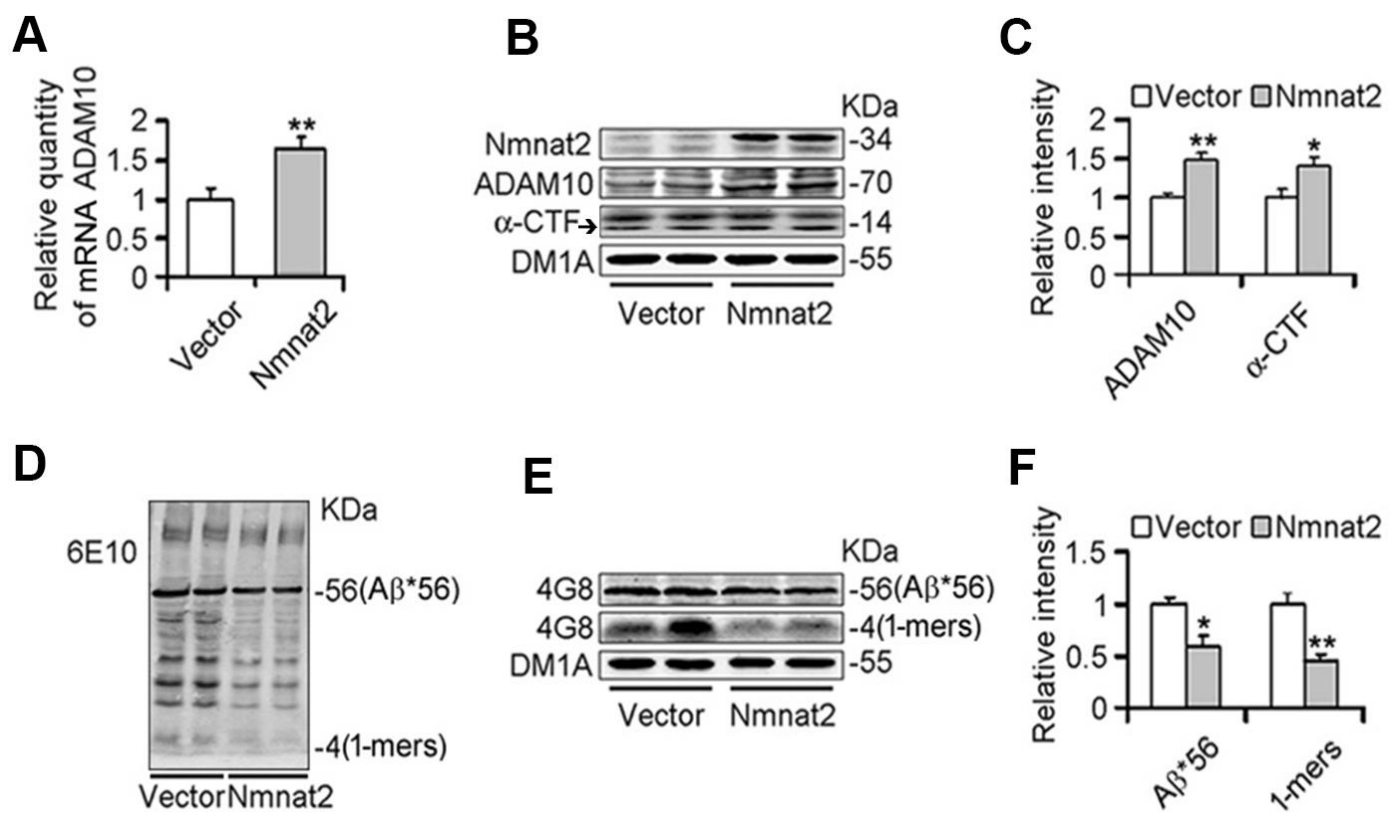
**Over-expression of Nmnat2 suppresses Aβ productions and up-regulates ADAM10 in N2a/APPswe cells.** N2a/APPswe cells were transfected with Flag-Nmnat2 (Nmnat2) or the empty vector for forty eight hours, and then mRNA level of ADAM10 and protein levels of ADAM10, α-CTF, and Aβ were detected by real-time PCR (**A**), western blot (**B**, **D**, **E**), and quantitative analysis (**C**, **F**). The data were representative of at least three independent experiments and expressed as means ± S.D.. *P < 0.05, **P < 0.01 vs control.

To further determine whether Nmnat2 over-expression causes the reduction of Aβ productions and increases ADAM10 expression, we transiently transfected the Nmnat2 plasmid in N2a/APPswe cells for 48 hours. Strikingly, the mRNA (Figure 2A) and protein levels of ADAM10 substantially increased in Nmnat2 over-expressing N2a/APPswe cells (Figure 2A–2C). In addition, we detected the productions of APP cleavage by α-secretase, a small fragment termed α-CTF and the large α-fragment ectodomain of APP (sAPPα), which are shown in Figure 2B, 2C and (Supplementary Figure 1A, 1B). The protein levels of α-CTF and sAPPα were significantly increased in Nmnat2 over-expressing cells compared to control. To eliminate the impact of overexposure of DM1A on data analysis, so we detected β-actin by western blot as the loading control. The images showed that the expression of DM1A was same as expression of β-actin in the same cell samples (Supplementary Figure 2A, 2B). Together, it is indicated that α-secretase is activated by Nmnat2.

**Figure 3:**
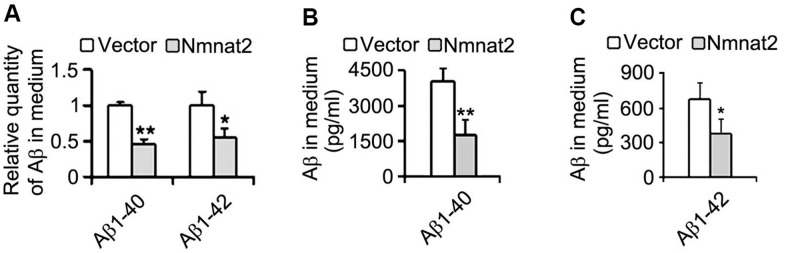
**Over-expression of Nmnat2 reduces Aβ in the medium of N2a/APPswe cells.** The medium of N2a/APPswe cells transfected with Flag-Nmnat2 (Nmnat2) or the empty vector for forty eight hours was measured for Aβ1-40 and Aβ1-42 by using ELISA and quantitative analysis (**A**–**C**). The data were representative of at least three independent experiments and expressed as means ± S.D.. *P < 0.05, **P < 0.01 vs control.

We next measured Aβ productions including soluble Aβ and its oligomers. Our results showed that soluble Aβ, its oligomers and Aβ^*^56-kDa were markedly reduced in N2a/APPswe cells with over-expression of Nmnat2 compared with control cells (Figure 2D–2F). To further determine whether over-expression of Nmnat2 suppresses Aβ production in N2a/APPswe cells, the mediums of N2a/APPswe cells transiently transfected the Nmnat2 plasmid or empty vector were collected and measured the levels of Aβ1-40 and Aβ1-42 by ELISA. The Aβ levels were obviously decreased in the medium of N2a/APPswe cells with Nmnat2 plasmid cDNAs compared with the medium of N2a/APPswe cells with Nmnat2 empty vector (Figure 3A–3C). In short, the above findings suggested that over-expression of Nmnat2 reduces Aβ production and up-regulates the expression of ADAM10.

### Nmnat2 activates AMPK by elevating the ratio of NAD^+^/NADH

**Figure 4:**
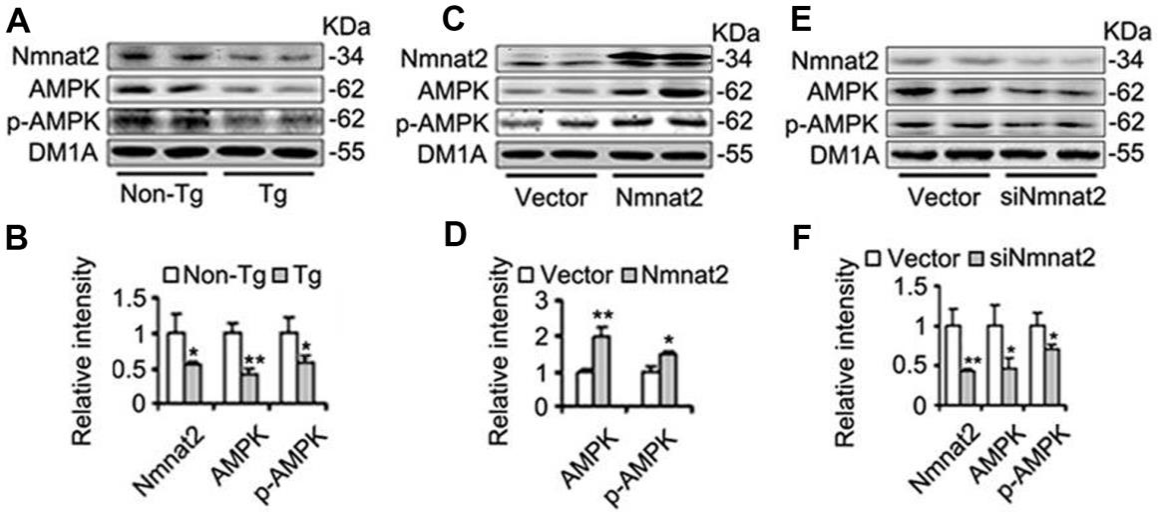
**Nmnat2 enhances expression of AMPK and p-AMPK.** (**A**, **B**) The cortex extracts were prepared from 10 months old Tg2576 mice (Tg) and the age-matched littermates (Non-Tg), then Nmnat2, AMPK and p-AMPK were measured by western blot (**A**) and quantitative analysis (**B**) (n=5 for each group). The data were expressed as means ± S.D. *P < 0.05, **P < 0.01 vs control. (**C**, **D**) N2a/APPswe cells were transfected with Flag-Nmnat2 (Nmnat2) or the empty vector for forty eight hours, and then Nmnat2, AMPK and p-AMPK were detected by western blot (**C**), quantitative analysis (**D**). (**E**, **F**) N2a/APPswe cells were transfected with shRNA-Nmnat2 (siNmnat2) or the scrambled shRNA control (Vector) for forty eight hours, and then Nmnat2, AMPK and p-AMPK were also detected by western blot (**E**), quantitative analysis (**F**). The data were representative of at least three independent experiments and expressed as means ± S.D.. *P < 0.05, **P < 0.01 vs control.

**Figure 5:**
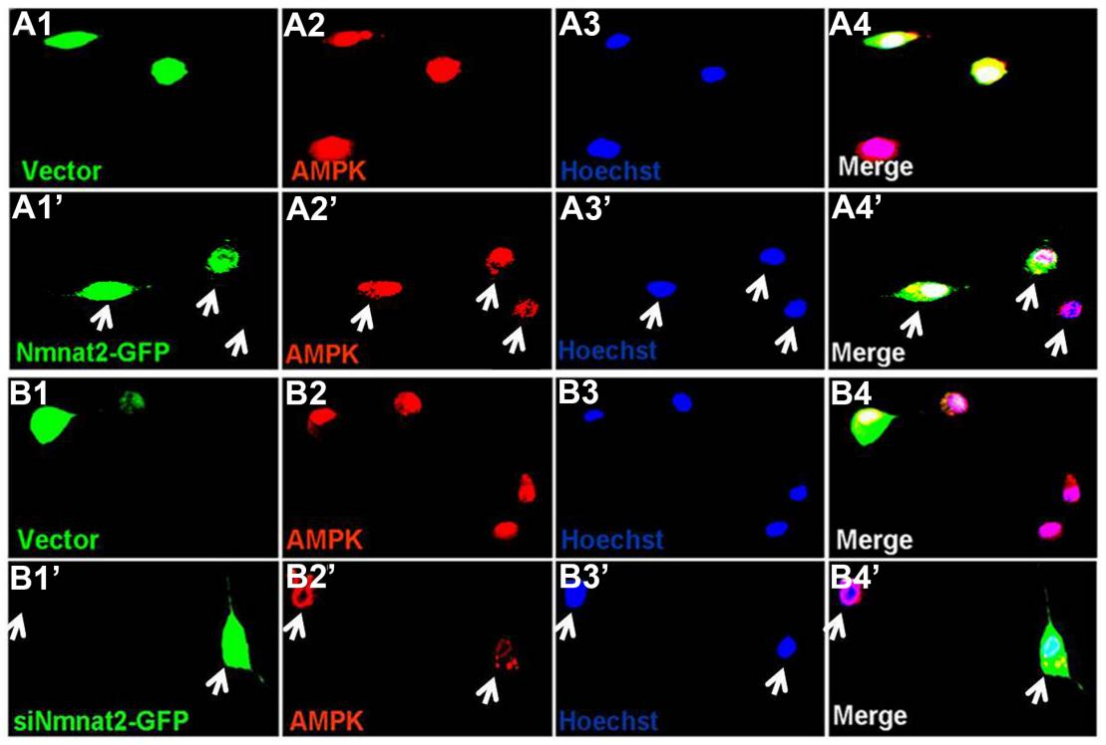
**Nmnat2 up-regulates AMPK in N2a/APPswe cells.** (**A1**–**A4ʹ**) N2a/APPswe cells were transfected with the empty vector (**A1**–**A4**) or Flag-Nmnat2 (Nmnat2) (**A1ʹ**–**A4ʹ**) for forty eight hours, and then AMPK was detected by immunofluorescence (IF), Nmnat2 over-expressing increased the fluorescence intensity of AMPK as shown by the white arrow (**A1ʹ**–**A4ʹ**). (**B1**–**B4ʹ**) N2a/APPswe cells were transfected with the scrambled shRNA control (Vector) (**B1**–**B4**) or shRNA-Nmnat2 (siNmnat2) (**B1ʹ**–**B4ʹ**) for forty eight hours, and then AMPK was detected by IF, knockdown of Nmnat2 decreased the fluorescence intensity of AMPK as shown by the white arrow (**B1ʹ**–**B4ʹ**). The data were representative of at least three independent experiments.

AMP-activated protein kinase (AMPK), a master regulator of cellular energy homeostasis [51], is potentially implicated in the pathogenesis of AD [52, 53]. The levels of AMPK and phosphorylated AMPK at Thr172 (p-AMPK, an active form) were measured by western blot in 10-month old Tg2576 mice and the age-matched wild type littermates. We found that the reduction of AMPK and p-AMPK (Figure 4A, 4B) was accompanied with the decrease of Nmnat2 in 10-month old Tg2576 mice. However, how Nmnat2 to regulate activity of AMPK needs to be further determined. So the Nmnat2 plasmid and empty vector were transfected into N2a/APPswe cells for 48 hours respectively, then the levels AMPK and p-AMPK were checked by western blot. The results showed that levels of AMPK and p-AMPK had a remarkable elevation in N2a/APPswe cells of over-expressing Nmnat2 compared to control cells (Figure 4C, 4D). To further confirm that the Nmnat2 is required for AMPK activity, we constructed shRNA plasmid of Nmnat2 (siNmnat2), and then the shRNA plasmid of Nmnat2 or the scrambled shRNA control (Vector) was transfected into N2a/APPswe cells for 48 hours. We found that the expression of endogenous Nmnat2 protein was efficaciously inhibited by siNmnat2 (reduction of 52.8%) (Figure 4E, 4F). Simultaneously, the levels of AMPK and p-AMPK were obviously decreased after normalization against DM1A (Figure 4E, 4F). In addition, we also employed immunofluorescence assays to further investigate the relationship between Nmnat2 and AMPK in N2a/APPswe cells through transiently transfecting the Nmnat2 and siNmnat2 plasmid cDNAs, respectively. We observed that the fluorescence intensity of AMPK was significantly elevated in N2a/APPswe cells with over-expressing Nmnat2 (Figure 5A1ʹ–5A4ʹ) compared with control cells (Figure 5A1–5A4). Conversely, the AMPK fluorescence intensity was substantially lower in N2a/APPswe cell transfected by siNmnat2 (Figure 5B1ʹ–5B4ʹ) than that in control cells (Figure 5B1–5B4). These results indicate that Nmnat2 up-regulates the expression and activity of AMPK *in vitro* and *in vivo*.

**Figure 6:**
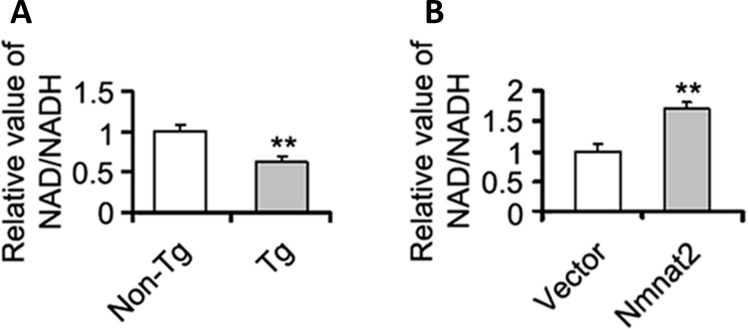
**Nmnat2 up-regulates the ratio of NAD^+^/NADH *in vitro* and *in vivo*.** (**A**) The cortex extracts were prepared from 10 months old Tg2576 mice (Tg) and the age-matched littermates (Non-Tg), then the ratio of NAD^+^/NADH was measured by NAD^+^/NADH quantification kit (**A**) (n=5 for each group). The data were expressed as means ± S.D. **P < 0.01 vs control. (**B**) N2a/APPswe cells were transfected with Flag-Nmnat2 (Nmnat2) or the empty vector for forty eight hours, and then the ratio of NAD^+^/NADH was detected by NAD^+^/NADH quantification kit (**B**). The data were representative from three independent experiments at least and expressed as means ± S.D.. **P < 0.01 vs control.

The mechanism of Nmnat2 regulating AMPK needs to be further determined. Studies have shown that a high intracellular NAD^+^/NADH ratio affects AMPK activity [54, 55]. Nmnat2 is a key enzyme to synthesize NAD^+^ using NMN and ATP [56, 57], and NAD^+^ is a cofactor required for glycolysis and tricarboxylic acid cycle, and involved in energy metabolism [58]. So we speculated that Nmnat2 activates AMPK via the ratio of NAD^+^/NADH. We detected the intracellular NAD^+^/NADH ratio in the cortex in 10-month old Tg2576 mice. There was a significant decrease of NAD^+^/NADH ratio in 10-month old Tg2576 mice compared to the age-matched wild type littermates (Figure 6A), which was positive correlation with the activity of AMPK. In contrast, we also measured the ratio of NAD^+^/NADH in N2a/APPswe cells transiently transfected by the Nmnat2 plasmid. The levels of AMPK and p-AMPK were clearly enhanced (Figures 4C, 4D, 5A1ʹ–5A4ʹ), and marked increased in the ratio of NAD^+^/NADH compared to control cells (Figure 6B), suggesting that Nmnat2 activating AMPK is dependent on ratio of NAD^+^/NADH.

### Nmnat2 attenuates Aβ production and up-regulates ADAM10 in AMPK activity-dependent manner

**Figure 7:**
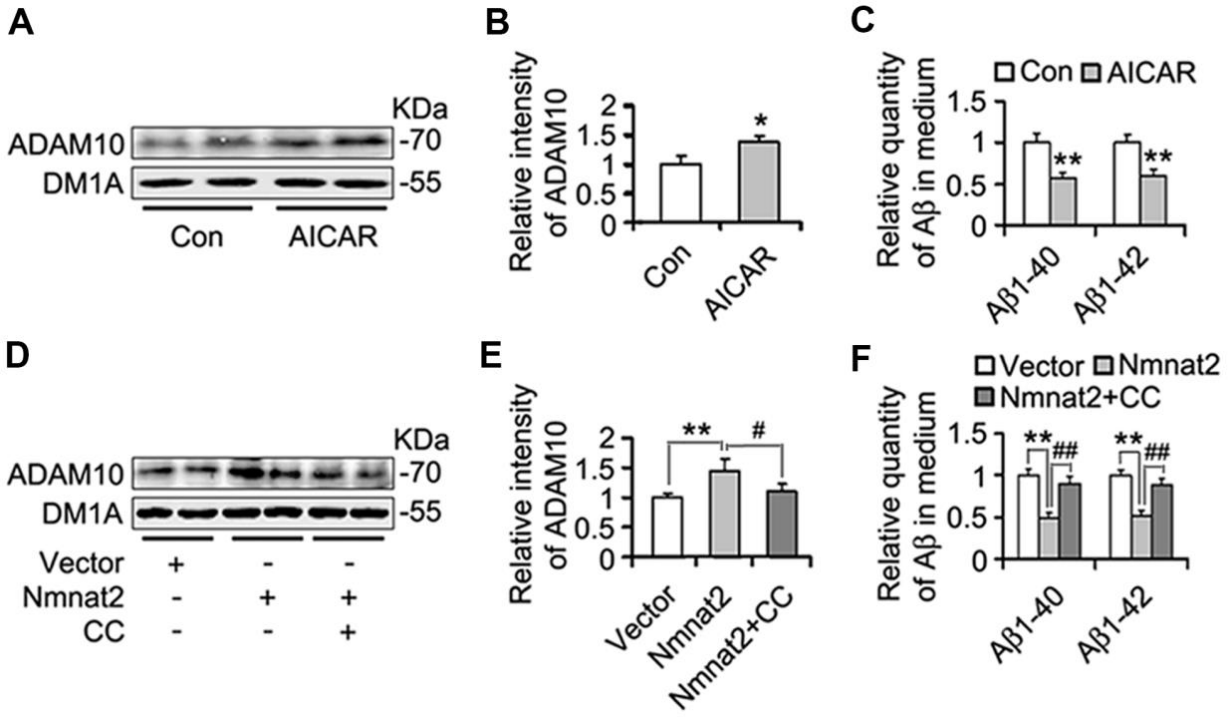
**Nmnat2 attenuates amyloidogenesis and up-regulates ADAM10 in AMPK activity-dependent manner in N2a/APPswe cells.** (**A**–**C**) N2a/APPswe cells were treated with 2 mM AICAR for twenty four hours, and then ADAM10 was detected by western blot (**A**) and quantitative analysis (**B**). The medium of N2a/APPswe cells treated with 2 mM AICAR for twenty four hours was measured for Aβ1-40 and Aβ1-42 by using ELISA (**C**). N2a/APPswe cells were transfected with Flag-Nmnat2 (Nmnat2) or empty vector for twenty four hours, and then treated with 20 μM Compound C (CC) for another twenty four hours. DMSO was used as the vehicle control of CC treatment. And then ADAM10 was measured by western blot (**D**) and quantitative analysis (**E**). The medium of N2a/APPswe cells transfected with Nmnat2 and CC treatment was detected for Aβ1-40 and Aβ1-42 by ELISA (**F**). The data were representative from three independent experiments at least and expressed as means ± S.D.. *P < 0.05, **P < 0.01 vs control; #*P* < 0.05, ##*P* < 0.01 vs Nmnat2.

Several studies have demonstrated that activation of AMPK alleviates Aβ pathogenesis and rescues synapse damage [59, 60]. We assumed that AMPK involves Nmnat2-mediated reduction of Aβ. To address this question, N2a/APPswe cells were treated with or without AICAR (2 mM) [61–63], an AMPK specific agonist for 24 hours. We observed that AICAR induced the expression of ADAM10 (Figure 7A, 7B) and reduced the productions of Aβ1-40 and Aβ1-42 (Figure 7C), suggesting that activating AMPK inhibits Aβ production through the up-regulation of ADAM10. To further determine that Nmnat2 attenuates Aβ production and up-regulates ADAM10 in AMPK activity-dependent manner. N2a/APPswe cells were transiently transfected by the Nmnat2 plasmid and then were treated with or without Compound C (20 μM, an AMPK specific inhibitor) [64–66] for another 24 hours. The results of western blot and ELISA assays showed that inhibiting AMPK activity with Compound C abolished the Nmnat2-induced up-regulation of ADAM10 levels (Figure 7D, 7E) and reduction of Aβ1-40 and Aβ1-42 (Figure 7F). These data provide the supporting evidence that Nmnat2 attenuates Aβ production and up-regulates the level of ADAM10 in AMPK activity-dependent manner.

## DISCUSSION

Our findings clearly indicated that the levels of α-secretase ADAM10, α-CTF and sAPPα of α-secretase cleavage are obviously decreased in Tg2576 mice. More interestingly, the Aβ productions including Aβ*56 (an Aβ dodecameric) and monomer, are also dramatically enhanced in the AD models, which have strong neurotoxicity and mainly cause damage of the ability of learning and recognition in the Tg2576 mice [48–50]. Our previous study has shown that expression of Nmnat2 is significantly reduced in Tg2576 mice [35], which the deficit of Nmnat2 is simultaneous with the increase of Aβ productions and reduction of ADAM10 in our present study. Moreover, the over-expression of Nmnat2 up-regulates ADAM10 and suppresses Aβ productions in N2a/APPswe cells. Nmnat2 appears to direct APP processing toward the α-secretase and away from the β-secretase, which results in a reduction at the productions of toxic Aβ peptides [67, 68]. Indeed, our data showed that Nmnat2 attenuates Aβ productions (including Aβ*56 and monomer) and up-regulates ADAM10 in AMPK activity-dependent manner. It is obvious that these data support an opinion, which Nmnat2 has neuroprotective effects on brain of AD patients and AD models [32, 33, 36, 69]. We demonstrated that Nmnat2 exerts its enzymatic function in AMPK activity-dependent manner to attenuate Aβ productions and up-regulate ADAM10 in this study.

Nmnat2 is a nicotinamide adenine dinucleotide (NAD^+^) synthesizing enzyme. Increasing evidence has shown that the loss of Nmnat2 leads to mitochondrial impairment as well as the fall in its substrate product NAD^+^ [24, 70, 71]. NAD^+^ is a crucial redox cofactor for metabolism and ATP production, and it is also a key substrate for several families of enzymes in health span and longevity [58, 72–74]. In current study, the decrease of NAD^+^/NADH ratio is related to the reduction of Nmnat2 expression in Tg2576 mice. Over-expression of Nmnat2 increases the NAD^+^/NADH ratio in N2a/APPswe cells, which is positive correlation with the NAD^+^/NADH ratio. However, the mechanism of Nmnat2 and its substrate product NAD^+^ regulating APP protein is unexplained.

AMPK, acting as an energy and nutrient sensor, coordinates an integrated signaling network, which constitutes metabolic pathways and involves AD pathogenesis [53, 75, 76]. Studies have found that a high intracellular NAD^+^/NADH ratio affects AMPK activity [54, 55]. In this study, we found that levels of AMPK and p-AMPK (Thr 172, active form) are lower in Tg2576 mice than in that of WT mice, which variation tendency of AMPK and p-AMPK are the same as Nmnat2 and ADAM10 in Tg2576 mice. And Nmnat2 positively regulates AMPK activity in N2a/APPswe cells with western blot and immunofluorescence by genetic manipulation*.* Therefore, we think that Nmnat2 adjusting AMPK activity is related to the increase of NAD^+^/NADH ratio, which is similar to previous results in vascular smooth [54, 55]. Growing evidence has shown that increase of ADAM10 levels will benefit for reduction of Aβ productions in AD-like models [13, 21, 77], which is similar to our study. Interestingly, we found that over-expression of Nmnat2 also enhances expression of ADAM10 and reduces Aβ1-40/1-42 in N2a/APPswe cells. The above data indicate that both of Nmnat2 and AMPK can activate ADAM10 and reduce Aβ production.

Recent studies have revealed a key neuronal maintenance and protective function for Nmnat [26–29]. Nmnat protects neurons through multiple functions such as NAD^+^ synthesizing enzymes and chaperones etc [24, 29, 31]. Loss and mutations in Nmnat are associated with human disease [78, 79]. Some studies suggest that AMPK may increase the elimination of the Aβ peptide via the induction of autophagy and neuronal cholesterol and sphingomyelin levels or other mechanisms [59, 63, 80, 81]. Our study demonstrate that activating AMPK with its specific agonist (AICAR) up-regulates the level of ADAM10 (a form of α-secretase activity) and reduced Aβ1-40/1-42 in N2a/APPswe cells. It is suggested that AMPK as a upstream molecule of ADAM10 activates its enzyme activity and inhibits Aβ productions. The reduction of Aβ1-40/1-42 and increase of ADAM10 could be induced by the over-expression of Nmnat2, which was blocked by Compound C (an AMPK specific antagonist) in N2a/APPswe cells. These data indicated that Nmnat2 activates α-secretase ADAM10 and inhibits Aβ productions in AMPK activity-dependent manner, which suggests that a new mechanism of Nmnat2 plays neuroprotective role in brain of AD models. An unavoidable issue rises. How does AMPK regulate ADAM10? Studies show that the intervention of AMPK/Sirt1 signaling pathway may improve neuropathological defects in Alzheimer’s disease [82, 83]. Previous reports have suggested that PKC activation may cause alterations in subcellular localization of ADAM10 and regulate secretion of amyloid precursor protein [84, 85]. Whether AMPK acts on ADAM10 through its direct kinase effect or AMPK-PKC pathway should be explored in future.

**Figure 8:**
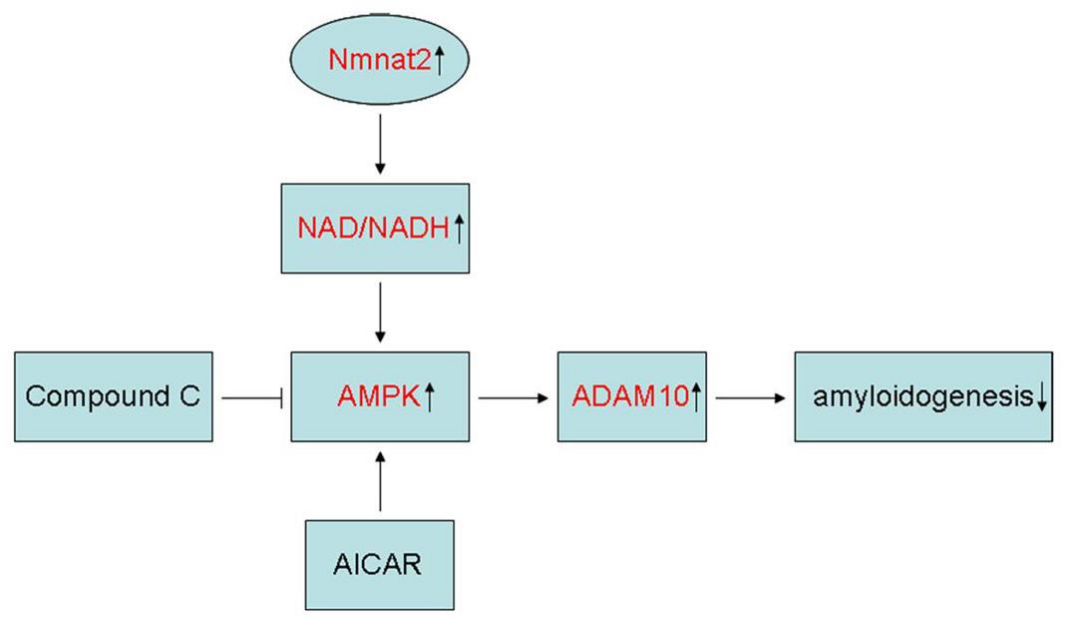
**Schematic representation of Nmnat2 attenuating amyloidogenesis.** Nmnat2 attenuates amyloidogenesis and up-regulates ADAM10 by increasing NAD^+^/NADH ratio in AMPK activity-dependent manner.

In summary, the present study showed that Nmnat2 attenuates amyloidogenesis and up-regulates ADAM10 by increasing NAD^+^/NADH ratio in AMPK activity-dependent manner (Figure 8). At the same time, it is also revealed that Nmnat2 might become a new target by increasing the activity of AMPK and ADAM10 to prevent Aβ generation in AD.

## Supplementary Material

Supplementary Figures
