# Supplementary material for: Nmnat2 attenuates amyloidogenesis and up-regulates ADAM10 in AMPK activity-dependent manner
Source: Aging (Albany NY). 2021 Oct 13;13(20):23620–36. doi: 10.18632/aging.203634 (PMC8580354; doi:10.18632/aging.203634)
Supplement: Supplementary Figures [file aging-13-203634-s001.pdf]

## SUPPLEMENTARY FIGURES

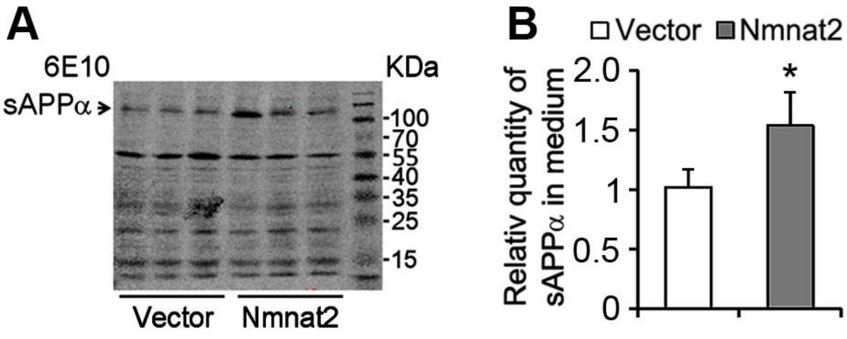


**Supplementary Figure 1. Over-expression of Nmnat2 increases sAPPα in the medium of N2a/APPswe cells.** The medium of N2a/APPswe cells transfected with Flag-Nmnat2 (Nmnat2) or the empty vector for forty eight hours was measured for sAPPα by western blot and quantitative analysis. (**A**, **B**) The data were representative of at least three independent experiments and expressed as means ± S.D.. *$P < 0.05$.

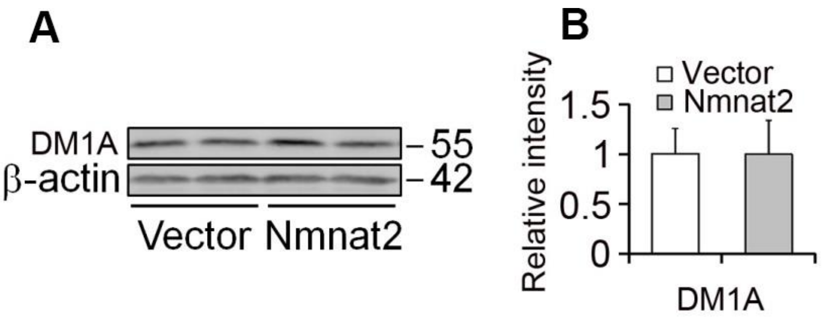


**Supplementary Figure 2. Expression of DM1A was the same as expression of β-actin in N2a/APPswe cells transfected with Flag-Nmnat2 (Nmnat2) or the empty vector.** N2a/APPswe cells were transfected with Flag-Nmnat2 (Nmnat2) or the empty vector for forty eight hours, and then DM1A and β-actin were detected by western blot (**A**) and quantitative analysis (**B**). The data were representative of at least three independent experiments and expressed as means ± S.D..
